# May-Thurner Syndrome Presenting with Pelvic Hemorrhage in the Setting of Blunt Trauma

**DOI:** 10.1155/2023/9003408

**Published:** 2023-06-05

**Authors:** Dan F. Laney, Alexandra H. Fairchild

**Affiliations:** Department of Radiology, Section of Interventional Radiology, Louisiana State University Health Sciences Center, 1542 Tulane Avenue, New Orleans, LA 70112, USA

## Abstract

May-Thurner Syndrome is a vascular condition in which chronic compression of the left common iliac vein by the overlying right common iliac artery causes impaired venous return from the left lower extremity as well as possible development of pelvic varicosities. The condition typically presents with acute left lower extremity deep vein thrombosis or with signs and symptoms of pelvic or lower extremity venous insufficiency. In our patient, however, the presenting symptom was hemorrhage of pelvic varicosities in the setting of extensive pelvic fractures sustained during a motor vehicle collision. Acute hemorrhage in the setting of pelvic fractures is typically associated with the need for arterial angiography and possible embolization. This patient was instead treated with venography and stenting of her May-Thurner lesion which resulted in the resolution of her bleeding pelvic varicosities and improvement in her pre-existing pelvic and lower extremity venous symptoms.

## 1. Introduction

May-Thurner Syndrome (MTS) is a well-known but uncommon vascular condition that typically presents with acute left lower extremity deep vein thrombosis (DVT) or with signs and symptoms of pelvic or lower extremity venous insufficiency. Hemorrhage of large presacral varicosities in the setting of acute pelvic fractures caused by blunt force trauma is a highly atypical initial presenting symptom for MTS that, to these authors' knowledge, has not been previously described. This case report will describe an unusual initial presentation of MTS in the setting of blunt trauma before discussing more typical presentations of MTS while reinforcing that, irrespective of presentation, the techniques used to diagnose and treat MTS remain the same.

## 2. Clinical Case

**Figure 1:**
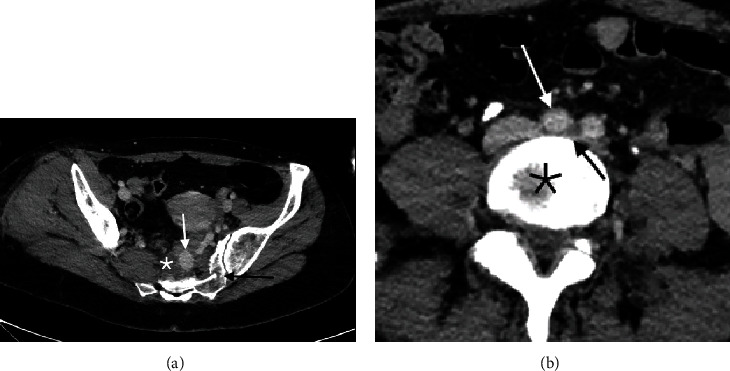
(a) Image from CT abdomen pelvis with contrast demonstrating pelvic varicosities as well as a large pseudoaneurysm (white arrow) surrounded by hematoma (star) anterior to a fractured sacrum (black arrow). (b) Additional CT image demonstrating compression of the left common iliac vein (black arrow) between the overlying right common iliac artery (white arrow) and the underlying vertebral body (star), typical of MTS anatomy.

A 41-year-old female was transferred from an outside hospital to the emergency department of a level 1 trauma center following a motor vehicle collision in which she was a restrained passenger in a car struck from behind by a semitrailer truck. She was hemodynamically stable upon presentation. Injuries described on imaging obtained both at the referring hospital and the level 1 trauma center included bilateral internal carotid artery dissections, multiple right-sided rib fractures, extensive pelvic fractures including multiple fractures of the left hemisacrum, and fracture of the proximal right femur. Computed tomography (CT) of the abdomen and pelvis with contrast also demonstrated a hematoma anterior to the sacrum and raised the possibility of active hemorrhage from an arterial pseudoaneurysm. Due to the concern that the patient could be actively bleeding, interventional radiology (IR) was consulted to evaluate for angiography and possible embolization, an intervention that is often utilized to treat acute arterial hemorrhage in the setting of traumatic pelvic fractures. After evaluation by IR, emergent intervention was deferred given her ongoing hemodynamic stability. When reviewing the patient's abdominopelvic CT, the IR service noted that the presacral hematoma surrounded a large venous pseudoaneurysm arising from large varicosities originating from the left internal iliac vein (Figure 1). Also appreciated was significant compression of the left common iliac vein between the overlying right common iliac artery and the fifth lumbar vertebral body, consistent with a possible May-Thurner lesion that could be the etiology of the patient's large pelvic varicosities (Figure 1). The CT did not demonstrate evidence of gonadal vein insufficiency or a proximal obstruction such as nutcracker syndrome.

**Figure 2:**
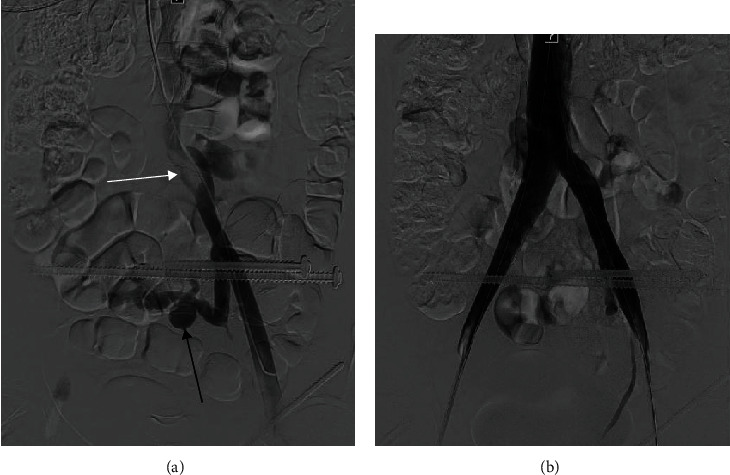
(a) Digital subtraction venography image demonstrating compression and narrowing of the left common iliac vein (white arrow) and a large venous pseudoaneurysm arising from pelvic varicosities (black arrow). These findings correspond with those seen on CT in Figure 1. (b) A digital subtraction venography image demonstrating the anatomic flow of contrast through the bilateral “kissing” iliac vein stents with resolution external compression of the left common iliac vein and associated narrowing. Also note that the pelvic varicosities and associated pseudoaneurysm are no longer visualized.

Following operative fixation of her pelvic and femur fractures by orthopedic surgery, her hemoglobin declined rapidly from 13.5 gm/dl to 7.1 gm/dl. The patient was subsequently transfused with 2 units of packed red blood cells, 1 unit of platelets, and 1 unit of cryoprecipitate, and her hemoglobin level stabilized. While some of this drop was thought to be attributable to blood loss during surgery, the possibility of active hemorrhage in the pelvis was again raised, perhaps from an arterial pseudoaneurysm. The IR service was reconsulted to evaluate for angiography and embolization. Given that the abnormal findings on her CT were thought by the IR service to be venous in etiology and there was concern that persistent venous hypertension within the varicosities and the associated pseudoaneurysm could predispose her to recurrent bleeding in the future, she was first taken for angiographic evaluation of her pelvic veins. Venography demonstrated apparent external compression of the left common iliac vein with retrograde flow of contrast down the left internal iliac vein and into the large presacral varicosities (Figure 2). A large venous pseudoaneurysm was also noted arising from the varicosities, correlating with findings on CT. No active hemorrhage was identified. Intravascular ultrasound (IVUS) examination confirmed hemodynamically significant external compression of the left common iliac vein, consistent with findings on both CT and venography. The area of narrowing of the left common iliac vein was serially dilated with 8 mm, 10 mm, and 12 mm diameter high pressure angioplasty balloons in order to decrease the pressure within the varicosities. Stenting was deferred at that time as therapeutic anticoagulation, which would be necessary following stent placement, was felt to be contraindicated due to her recent hemorrhage, extensive orthopedic injuries, and the possibility that she might need further surgery in the near future.

During the following week, the patient's clinical condition improved, she remained hemodynamically stable without further evidence of bleeding, and she was given medical clearance to initiate therapeutic anticoagulation. Due to a concern for possible rebleeding in the future, she was returned to the angiography suite with the IR service for stenting of her left common iliac vein compression to decompress her large pelvic varicosity. Two 14 mm bare metal self-expanding stents were deployed in a “kissing” fashion, extending from the inferior vena cava (IVC), through the bilateral common femoral veins, and into the proximal portion of the bilateral external iliac veins. Completion angiography demonstrated laminar flow of contrast through the stented veins and into the IVC with accompanying cessation of flow through the pelvic varicosities including the previously noted venous pseudoaneurysm (Figure 2). Following iliac vein stenting, the patient was prescribed enoxaparin for two weeks, clopidogrel for three months, and acetylsalicylic acid for life. She was compliant with this regimen and tolerated it well.

The patient was followed up in the IR clinic two weeks later. At that time, she noted that before the motor vehicle collision, she did have chronic pelvic and lower extremity heaviness with prolonged standing. She stated that her pelvic symptoms had improved following the intervention. During that clinic visit, she was unable to discern if her lower extremity symptoms had improved due to the continued sequela of her traumatic injuries sustained during the accident. When seen again in the IR clinic one month later, she stated that her lower extremity symptoms had improved as well.

### 2.1. Ethical Approval to Publish

This case report was prepared in accordance with the requirements of the HIPAA privacy regulations. Approval is not required for case reports such as this by our institutional IRB

## 3. Discussion

MTS occurs due to compression of the left common iliac between the overlying right common iliac artery and the underlying 5th lumbar vertebral body [1]. The anatomy of MTS was not described until 1957, when May and Thurner described fibrous bands of tissue they called “spurs” in the common iliac veins of 22% of 430 cadavers dissected [2]. It is currently estimated that up to ⅓ of humans have hemodynamically significant compression of their left iliac vein by the overlying right iliac artery [1]. Despite this, the prevalence of MTS in which patients have symptoms secondary to this anatomic finding is much lower. The exact prevalence of MTS is difficult to determine owing to the assumption that symptoms secondary to MTS are likely underreported or misattributed to other medical conditions [3].

The classic presenting symptom of MTS is left lower extremity DVT. MTS is thought to be responsible for 2-5% of all DVT [3]. However, nonthrombotic symptoms of developing venous insufficiency can also signify the presence of the syndrome. Such symptoms include pelvic or lower extremity pain or heaviness, leg swelling, skin changes, varicose veins, or even venous ulcers in advanced disease [1].

The gold standard for the diagnosis of MTS is venography and IVUS examination [1]. Venography allows for a dynamic examination of venous blood flow including the identification of any collateral pathways of flow. IVUS examination allows for the identification of the exact location of the area of venous narrowing and the quantification of the percentage of narrowing by comparing the area of the narrowed segment to an area of an adjacent normal vein. A stenosis of greater than 50% is generally considered hemodynamically significant.

The typical treatment for MTS is stenting of the region of the compressed vein as stenting of iliac vein compression is recommended by both the Society of Interventional Radiology [4] and the Society for Vascular Surgery [5]. Stenting of the hemodynamically significant stenosis results in normal, laminar flow through the external and common iliac vein and cessation of flow through venous collateral or varicosities, as was seen in our patient. Patients who undergo stenting typically experience improvement or resolution of their MTS associated venous symptoms.

While the presenting symptom of MTS in this patient was highly atypical, the diagnosis and treatment of the underlying process were the same as they would be in any patient thought to have MTS. Further, the improvement in the patient's venous insufficiency symptoms following treatment was typical of patients treated appropriately for MTS.

## 4. Limitation

The primary limitation of this manuscript is that it is a case report involving our experience with a single patient at a single center.

## 5. Conclusion

MTS typically presents with left lower extremity DVT or signs and symptoms of pelvic or lower extremity venous insufficiency. Hemorrhage in the setting of blunt trauma, as in the case described, is a highly unusual presenting symptom for MTS. Despite this atypical presentation, diagnosis of the condition with venography and treatment with iliac vein stenting was the same.

## Data Availability

No data was used.

## References

[1] Knuttinen M. G., Naidu S., Oklu R. (2017). May-Thurner: diagnosis and endovascular management. *Cardiovascular Diagnosis and Therapy*.

[2] May R., Thurner J. (1957). The cause of the predominantly sinistral occurrence of thrombosis of the pelvic veins. *Angiology*.

[3] Harbin M. M., Lutsey P. L. (2020). May‐Thurner syndrome: history of understanding and need for defining population prevalence. *Journal of Thrombosis and Haemostasis*.

[4] Vedantham S., Desai K. R., Weinberg I. (2023). Society of Interventional Radiology Position Statement on the endovascular management of acute iliofemoral deep vein thrombosis. *Journal of Vascular and Interventional Radiology*.

[5] Meissner M. H., Gloviczki P., Comerota A. J. (2012). Early thrombus removal strategies for acute deep venous thrombosis: clinical practice guidelines of the Society for Vascular Surgery and the American Venous Forum. *Journal of Vascular Surgery*.

